# DNA Methylation profiles as predictors of recurrence in non muscle invasive bladder cancer: an MS-MLPA approach

**DOI:** 10.1186/1756-9966-32-94

**Published:** 2013-11-19

**Authors:** Valentina Casadio, Chiara Molinari, Daniele Calistri, Michela Tebaldi, Roberta Gunelli, Luigi Serra, Fabio Falcini, Chiara Zingaretti, Rosella Silvestrini, Dino Amadori, Wainer Zoli

**Affiliations:** 1 Biosciences Laboratory, Istituto Scientifico Romagnolo per lo Studio e la Cura dei Tumori (IRST) IRCCS, Via P. Maroncelli 40, Meldola 47014, Italy; 2 Department of Urology, Morgagni Pierantoni Hospital, Forli, Italy; 3 Pathology Unit, Morgagni Pierantoni Hospital, Forlì, Italy; 4 National Institute of Molecular Genetics, Milan, Italy; 5 Department of Medical Oncology, IRST IRCCS, Meldola, Italy

**Keywords:** Non muscle invasive bladder cancer (NMIBC), Gene methylation profile, Recurrence

## Abstract

**Background:**

Although non muscle invasive bladder cancer (NMIBC) generally has a good long-term prognosis, up to 80% of patients will nevertheless experience local recurrence after the primary tumor resection. The search for markers capable of accurately identifying patients at high risk of recurrence is ongoing. We retrospectively evaluated the methylation status of a panel of 24 tumor suppressor genes (TIMP3, APC, CDKN2A, MLH1, ATM, RARB, CDKN2B, HIC1, CHFR, BRCA1, CASP8, CDKN1B, PTEN, BRCA2, CD44, RASSF1, DAPK1, FHIT, VHL, ESR1, TP73, IGSF4, GSTP1 and CDH13) in primary lesions to obtain information about their role in predicting local recurrence in NMIBC.

**Methods:**

Formaldehyde-fixed paraffin-embedded (FFPE) samples from 74 patients operated on for bladder cancer were analyzed by methylation-specific multiplex ligation-dependent probe amplification (MS-MLPA): 36 patients had relapsed and 38 were disease-free at the 5-year follow up. Methylation status was considered as a dichotomous variable and genes showing methylation ≥20% were defined as “positive”.

**Results:**

Methylation frequencies were higher in non recurring than recurring tumors. A statistically significant difference was observed for HIC1 (*P* = 0.03), GSTP1 (*P* = 0.02) and RASSF1 (*P* = 0.03). The combination of the three genes showed 78% sensitivity and 66% specificity in identifying recurrent patients, with an overall accuracy of 72%.

**Conclusions:**

Our preliminary data suggest a potential role of HIC1, GSTP1 and RASSF1 in predicting local recurrence in NMIBC. Such information could help clinicians to identify patients at high risk of recurrence who require close monitoring during follow up.

## Background

Although superficial bladder cancer generally has a good long-term prognosis, up to 80% of patients will have local recurrence within 5 years of the primary tumor resection [1]. After transurethral resection of bladder cancer (TURB), standard follow up involves numerous cystoscopies with consequently high healthcare costs and low patient compliance. Multiplicity, tumor size and prior relapse rate are the only recurrence-related parameters currently available for monitoring patients with bladder cancer [1], but such information would not seem to be accurate enough to ensure an adequate follow-up of individuals with stage Ta-T1 non muscle invasive bladder cancer (NMIBC). It would thus be extremely useful for clinicians to have new biological markers that can predict recurrence more accurately.

The role of epigenetic alterations in the carcinogenesis of solid tumors has been intensively investigated over the last ten years [2,3]. DNA methylation at CpG rich regions often occurs at tumor suppressor gene promoters, frequently producing a reduction in the expression of target genes. An increasing number of papers are being published on the role of gene methylation and its potential clinical application in human tumors [4]. Methylation seems to be an early event in the development of a number of solid tumors including bladder cancer [5,6] and can thus be regarded as an early sign of cancer before the disease becomes muscle-invasive. Methylated tumor suppressor genes such as APC, RARB2, BRCA1 have recently been indicated as valid diagnostic markers for NMIBC [7-10]. A number of papers have also focused on the role of methylation as a prognostic marker, but it is not clear which methylated genes can accurately predict recurrence. Some studies have hypothesized hypermethylation of tumor suppressor genes, such as TIMP3, as a good prognostic marker [11,12], while others have indicated hypermethylated E-cadherin, p16, p14, RASSF1, DAPK, APC, alone or in different combinations, as potential markers of early recurrence and poor survival [13-15].

In the present study we evaluated the methylation status of a panel of 24 genes (TIMP3, APC, CDKN2A, MLH1, ATM, RARB, CDKN2B, HIC1, CHFR, BRCA1, CASP8, CDKN1B, PTEN, BRCA2, CD44, RASSF1, DAPK1, FHIT, VHL, ESR1, TP73, IGSF4, GSTP1 and CDH13) in superficial bladder cancer to determine their ability to predict recurrence. Although methylation of some of these genes has already been investigated in bladder cancer [11-15], its relevance as an indicator of recurrence has yet to be confirmed. We used the relatively new methodology of methylation specific multiplex ligation dependent probe amplification (MS-MLPA) to evaluate epigenetic gene profiles. This approach permits methylation analysis of multiple targets in a single experiment [16,17] and has been successfully used to evaluate the diagnostic or prognostic relevance of different markers in several tumor types such as lung [18], rectal [19], breast [20] and recently, bladder cancers [7,8].

## Methods

### Case series (retrospective cohort study)

Tissue samples from 74 patients (65 males, 9 females) submitted to transurethral resection of primary bladder cancer at the Department of Urology of Morgagni-Pierantoni Hospital in Forlì between 1997 and 2006 were used for the study. All samples were retrieved from the archives of the Pathology Unit of the same hospital. Median age of patients was 73 years (range 39–92): 31 were <70 years and 43 ≥70 years. On the basis of 2004 World Health Organization criteria, final diagnosis was low grade non muscle invasive bladder cancer (NMIBC) in 55 patients and high grade NMIBC in 19 patients. At a median follow up of 5 years 38 patients were still disease-free and 36 had experienced one or more episodes of local recurrence. In this retrospective study, the two subgroups (disease free or relapsed) of patients were equally distributed for sex, age, grade and stage (Table 1).

**Table 1:** Case series

	**Patients**	
	**Recurrent**	**Non recurrent**
**Sex**		
**Male**	33	32
**Female**	3	6
**Age**, **years**		
**<70**	19	12
**≥70**	17	26
**Grade**		
**Low**	27	28
**High**	9	10
**Stage**		
**Ta**	30	31
**T1**	6	7

All patients gave written informed consent for biological samples to be used for research purposes. The study protocol was reviewed and approved by the ‘*Area Vasta’ Istituto Scientifico Romagnolo per lo Studio e la Cura dei Tumori* (*IRST)* Ethics Committee.

### Macrodissection and DNA isolation

Five 5-μm-thick sections were obtained from each paraffin-embedded block. Macrodissection was performed on hematoxylin-eosin stained sections and only cancer tissue was used for DNA isolation. Genomic DNA was purified using QIAmp DNA FFPE Tissue (Qiagen, Milan), according to the manufacturer’s instructions.

DNA was also isolated from a human bladder cancer cell line (HT1376) using Qiamp DNA minikit (Qiagen, Milan, Italy), according to the manufacturer’s instructions.

### Methylation specific multiple ligation probe amplification (MS-MPLA)

**Figure 1:**
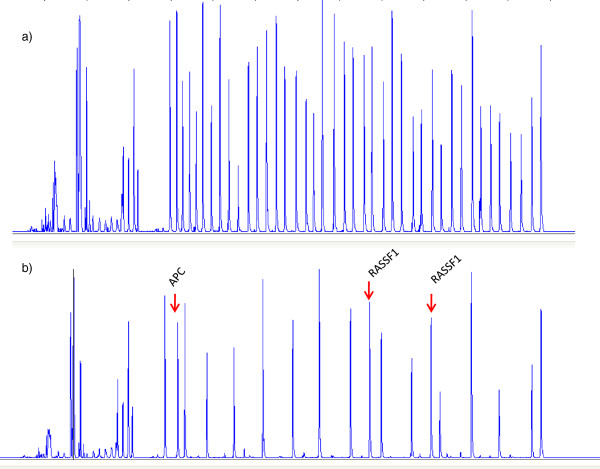
Electropherogram relating to a) undigested and b) digested HT1376 samples with methylation of APC and RASSF1 genes.

MS-MLPA was performed using at least 50 ng of genomic DNA dissolved in 1XTE buffer (Promega, Madison, WI, USA). DNA isolated from HT 1376 cell line was used as internal control for MS MLPA analysis (Figure 1). The methylation status of 24 tumor suppressor gene promoters was analyzed using the ME001C1 kit (MRC-Holland, Amsterdam, The Netherlands) (Table 2). Two different probes that recognize two different sites of the promoter region were used for genes RASSF1 and MLH. We excluded CDKN2B gene from the analysis because its probe is sensitive to improper Hha1 digestion in FFPE samples. In brief, DNA was denatured (10 min at 98°C) and cooled at 25°C, after which the probe mix was added to the samples and hybridization was performed by incubation at 60°C for 16–18 h. The reaction was divided equally in two vials, one for ligation and the other for ligation-digestion reaction for each tumor. We added a mix composed of Ligase-65 buffer, Ligase-65 enzyme and water to the first vial and a mix of Ligase-65 Buffer, Ligase 65 enzyme, Hha1 enzyme (Promega, UK) and water to the second. The samples were then incubated at 49°C for 30 min. At the end of the ligation and ligation-digestion reactions, samples were amplified by adding a mix of PCR buffer, dNTPs and Taq polymerase. The PCR reaction was performed under the following conditions: 37 cycles at 95°C for 30 sec, 60°C for 30 sec and 72°C for 60 sec. The final incubation was performed at 73°C for 20 min.

**Table 2:** Summary of gene function and chromosomal localization

**Gene**	**Function**	**Chromosomal localization**
TIMP metallopeptidase inhibitor 3 (TIMP3)	Invasion and metastasis	22q12.3
Adenomatous polyposis coli (APC)	WNT antagonist	5q22
cyclin-dependent kinase inhibitor 2A (CDKN2A)	Cell-cycle control gene	9p21
MutL homolog 1, colon cancer, nonpolyposis type 2 (MLH1)	DNA mismatch repair	3p22.1
Ataxia telangiectasia mutated (ATM)	Cell-cycle control gene	11q23
Retinoic acid receptor, beta (RARB)	Cell differentiation and proliferation	3p24.2
Hypermethylated in Cancer 1(HIC1)	Putative tumor suppressor gene	17p13.3
Checkpoint with forkhead and ring finger domains (CHFR)	Putative tumor suppressor gene	12q24.33
breast cancer 1, early onset (BRCA1)	Maintenant of genomic stability	17q21.31
Caspase 8, apoptosis-related cysteine peptidase (CASP8)	Apoptosis related gene	2q33.2
Cyclin-dependent kinase inhibitor 1B (CDKN1B)	Cell-cycle control gene	12p13.2
Phosphatase and tensin homolog (PTEN)	Cell-cycle regulation gene	10q23.3
Breast cancer 2, early onset (BRCA2)	Maintenance of genomic stability	13q12.3
CD44 molecule (Indian blood group) (CD44)	Cell-cell interaction mediator	11p12
Ras association (RalGDS/AF-6) domain family member 1 (RASSF1)	Putative tumor suppressor gene	3p21.3
Death-associated protein kinase1 (DAPK)	Apoptosis-related gene	9q34.1
Von Hippel-Lindau tumor suppressor (VHL)	Putative tumor suppressor gene	3p25
Estrogen receptor 1 (ESR1)	Cell differentiation and proliferation	6q25.1
Tumor protein p73 (TP73)	Apoptotic response to DNA damage	1p36.32
Fragile histidine triad gene (FHIT)	Putative tumor suppressor gene	3p14.2
Cell adhesion molecule 1 (IGSF4 (CADM1))	Cell adhesion related gene	11q23
Cadherin 13, H-cadherin (heart) (CDH13)	Cell invasion	16q23.3
Glutathione S-transferase pi 1 (GSTP1)	DNA damage repair gene	11q13

Amplification products were analyzed by ABI-3130 genetic Analyzer (Applied Biosystem, UK). Universally methylated and unmethylated genomic DNA was used as positive or negative control, respectively.

Electropherograms obtained were analyzed using Gene Mapper software (Applied Biosystem, UK) and the peak areas of each probe were exported to a home-made excel spreadsheet. In accordance with the manufacturer’s instructions, we carried out “intrasample data normalization” by dividing the signal of each probe by the signal of every reference probe in the sample, thus creating as many ratios per probe as there were reference probes. We then calculated the median value of all probe ratios per probe, obtaining the normalization constant (NC). Finally, the methylation status of each probe was calculated by dividing the NC of a probe in the digested sample by the NC of the same probe in the undigested sample, and by multiplying this ratio by 100 to have a percentage value, as follows:

$$\frac{\text{NC}\left(\text{digested}\;\text{sample}\right)}{\text{NC}\left(\text{undigested}\;\text{sample}\right)}\times 100$$

MS-MLPA technique reproducibility was assessed by performing three independent methylation profile analyses on a bladder cell line (HT1376). The methylation level for each gene was found to be the same in each experiment.

We considered the promoters showing a ratio ≥0.20 as methylated, while those with a ratio <0.20 were regarded as unmethylated. The cut-off was chosen on the basis of experiments performed on the bladder cancer cell line (HT1376) and on data from the literature [21,22]. We have also performed the analysis on some samples from healthy tissues, to confirm that the background noise was inferior to 0.20 cut-off, such excluding false positive results due to experimental procedure.

### Statistical analysis

Fisher’s exact test was used to compare the frequency of promoter methylation in the two subgroups: recurrent tumors versus non recurrent tumors. Methylation status was considered as a dichotomic variable and genes showing methylation ≥ 20% were classified as positive. A difference was considered significant if it showed a two-tailed *P* value ≤0.05. The genes showing a significant p value in Fisher’s exact test were used to analyze the methylator phenotype. Study endpoints were sensitivity (the proportion of recurrent cancer patients who were correctly identified by the test or procedures) and specificity (the proportion of non recurrent cancer patients who were correctly identified), with their 95% confidence intervals (CIs). We also evaluated overall accuracy, defined as the proportion of the total number of patients correctly identified by the test.

The student’s T test was used to assess the methylation index (MI), which was considered as a continuous variable. Logistic regression analysis was performed using the Epicalc of R to evaluate the performance of a panel of gene promoters (HIC1, RASSF1 and GSTP1) in discriminating between recurrent and non recurrent patients. We created logistic regression models with methylation levels of the three gene promoters (HIC1, RASSF1 and GSTP1). Probabilities were calculated as follows: *P* = exp ((Σ(b_i_x_i_) + c)/(1 + Σ(b_i_x_i_) + c), where p is the probability of each case, i = 1 to n; b is the regression coefficient of a given gene, x is the log2-transformed methylation level and c is a constant generated by the model. The ROCR package was used to obtain the ROC curves of the models and area under the curve (AUC) values. Recurrence-free survival was analyzed with the Log-rank test using SAS 9.3 software. All the molecular analyses were performed in a blind manner.

## Results

**Figure 2:**
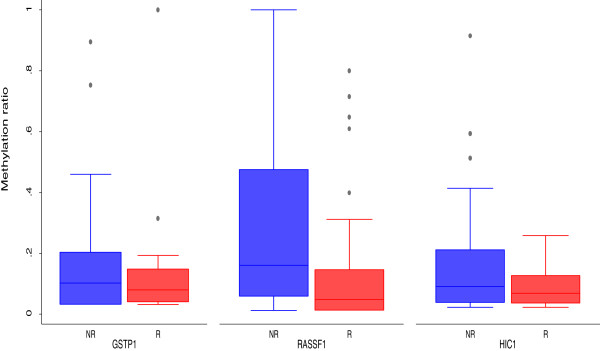
Methylation levels of the three significant genes (HIC1, RASSF1, GSTP1) showed as box plot.

MS-MLPA analysis was feasible in all samples. The methylation frequency in the overall series varied widely (1% to 50%) for the different genes (Table 3). A separate analysis as a function of recurrence showed lower gene methylation in recurring than non recurring tumors, with the exception of CDKN1B, FHIT and IGSF4 genes. However, a significant difference between recurrent and non recurrent tumors was only observed for GSTP1, HIC1 and RASSF1 locus 2 (Table 3), with lower methylation in relapsed than non relapsed patients (Figure 2). The methylation index (MI), evaluated as the number of methylated genes relative to the total number of analyzed genes, showed values from 0 to 0.68 in the overall series of 23 genes and a significantly lower median value in non recurrent (0.08) than recurrent (0.12) (*P* = 0.011) patients (Table 4). To reduce the complexity of the methodological approach, further analysis was limited to a series of 10 genes (GSTP1, HIC1, RASSF1-locus2, CD44,DAPK, RASSF1-locus1, TP73, BRCA1, ESR1, TIMP3) that proved significant or showed a trend towards significance (*P* values varying from 0.02 to 0.31). Again, a higher median MI was seen in patients who relapsed compared to those who did not (0 versus 0.2; *P* = 0.0007) (Table 4).

**Table 3:** Methylation frequencies of different genes in the overall series and in non recurrent or recurrent tumors

	**Frequency (%)**			
**Gene**	**Overall series (n = 74)**	**Non recurrent tumors (n = 38)**	**Recurrent tumors (n = 36)**	P value*
CD44	1	18	3	0.06
CASP8	1	3	0	1
MLH1 (locus 2)	1	3	0	1
PTEN	3	5	0	0.49
VHL	3	5	0	0.49
BRCA1	4	8	0	0.24
CHFR	4	5	3	1
ATM	5	8	3	0.62
BRCA2	5	8	3	0.62
CDKN1B	5	5	5	1
RARB	6	8	6	1
**HIC1**	**9**	**16**	**0**	**0.03**
FHIT	10	1	10	1
MLH1 (locus 1)	11	15	8	0.48
ESR1	12	16	6	0.26
TIMP3	13	18	8	0.31
TP73	14	19	8	0.19
CDKN2A	14	16	14	1
**GSTP1**	**15**	**26**	**5**	**0.02**
DAPK	17	24	8	0.11
IGSF4 (CADM1)	21	18	25	0.58
RASSF1 (locus 1)	23	29	14	0.16
APC	29	34	25	0.45
**RASSF1(locus2)**	**33**	**45**	**19**	**0.03**
CDH13	50	53	47	0.81

*Fisher’s exact test 2-tailed P value (difference between recurrent and non recurrent tumors). Significant genes are highlighted as bold data.

**Table 4:** Methylation index analyisis

	**Median value**			***P *** **value**
**Methylation index (MI)**	**Overall**	**Recurrence**	**No recurrence**	
23 Genes*	0.1	0.08	0.12	0.011
10 Genes**	0.2	0	0.2	0.0007

*MI = Number of methylated genes/number of analyzed genes.

**MI=number of methylated genes/ 10 genes (GSTP; HIC1; RASSF1 (LOCUS 1); RASSF1 (LOCUS 2); CD44; DAPK; TP73; BRCA1; ESR; TIMP3).

**Figure 3:**
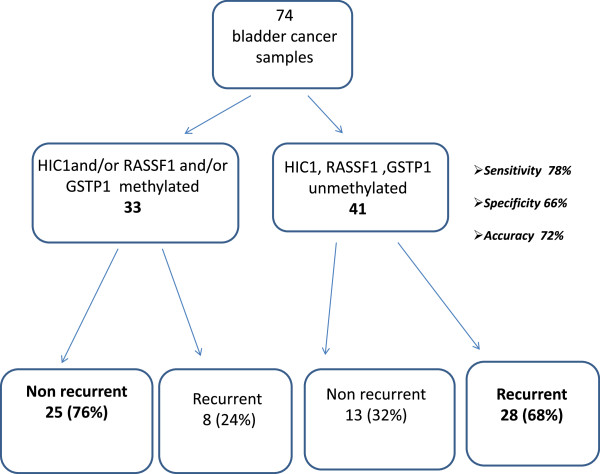
**Prognostic algorithm with the three significant genes (GSTP1, HIC1 and RASSF1).** Sensitivity was evaluated as the number of recurrent tumors with unmethylated *HIC1*, *RASSF1*, *GSTP1* relative to the total number of recurrent tumors analyzed. Specificity was evaluated as the number of non recurrent tumors with methylated phenotype relative to the total number of non recurrent tumors analyzed. Overall accuracy was calculated as the number of correctly classified tumors relative to the total number of analyzed tumors.

We constructed a prognostic algorithm with the 3 significant genes (GSTP1, HIC1 and RASSF1) considering two phenotypes: the “methylated phenotype” (MP) (samples with at least one of the three genes methylated), and the “unmethylated phenotype” (samples with none of the three genes methylated). Of the 33 patients with methylated phenotype, 25 (76%) were still disease-free and 8 (24%) had had at least one intravescical recurrence at a median follow up of 5 years (Figure 3). Conversely, of the 41 patients with unmethylated phenotype, 28 (68%) had relapsed within 5 years of surgery and 13 (32%) had remained disease-free. The three-gene panel showed 78% sensitivity in identifying recurrent tumors and 66% specificity, with an overall accuracy of 72%.

**Figure 4:**
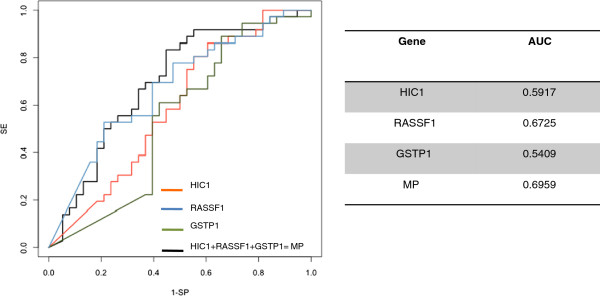
**ROC curves relating to the three significant genes (HIC1,RASSF1, GSTP1) analyzed singly or in combination.**

We also performed ROC curve analysis for the three significant genes, singly or in combination, considered as continuous variables. Resultant AUCs were 0.5917 for HIC1, 0.6725 for RASSF1 and 0.5409 for GSTP1, the best AUC (0.6959) reached for the combination of the three genes (Figure 4).

**Figure 5:**
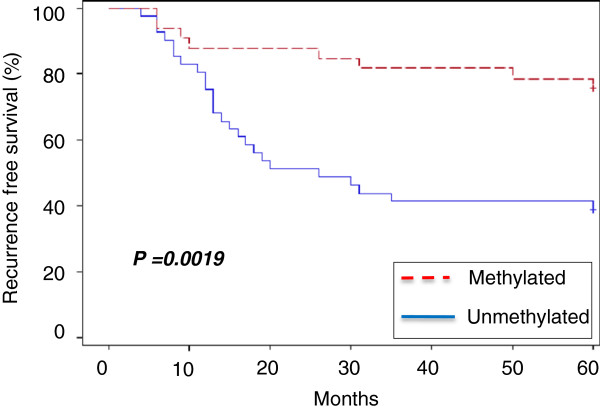
**Recurrence**-**free survival in patients with methylated phenotype (samples with at least one of the three significant genes methylated) or unmethylated phenotype (samples with none of the three genes methylated).**

Recurrence-free survival analysis of patients with methylated or unmethylated tumors highlighted a significantly higher recurrence-free survival (*P* = 0.0019) for those whose tumors showed the methylated phenotype (Figure 5).

The recurrence free survival analysis performed considering only the recurrent patients, showed that patients with unmethylated tumors had a lower median recurrent free survival time (14.5 months), with the respect to patients with methylated ones (18 months). However, the two subgroups are not equal distributed to give a statistical significant result (P = 0.9392, data not shown).

Multivariable analysis considering clinical and biological parameters (patient age and sex; tumor grade, stage and size; tumor multiplicity, methylated phenotype) showed that only age and methylated phenotype were independent predictors of recurrence. Specifically, patients under 70 years of age showed a higher probability of relapsing than older ones (*P* = 0.028) and their methylation phenotype was significantly predictive of recurrence (*P* < 0.0001).

## Discussion

The present study focused on evaluating the methylation status of tumor suppressor genes and on verifying its role in predicting recurrence of non muscle invasive bladder cancer (NMIBC). The MS-MLPA technique has the advantage of requiring only a small quantity of DNA, is capable of rapidly determining the methylation status of numerous genes in the same experiment, and has also been shown to work well in formalin-fixed paraffin-embedded samples. However, an important limitation of our study was the lack of a sufficient quantity of cancer tissue to confirm the methylation results using a second technique such as methylation specific PCR (MS PCR) or gene expression analyses.

In agreement with results from other studies [18], we found a positive correlation between gene methylation and lack of recurrence, highlighting that putative tumor suppressor genes do not always act as tumor suppressors but may actually have different biological functions.

Statistical analysis revealed 3 genes (HIC1, GSTP1, and RASSF1) capable of significantly predicting tumor recurrence. Their methylation was significantly indicative of a lack of recurrence at the 5-year follow up. The combined analysis of the three genes showed 72% accuracy in predicting recurrence or non recurrence.

HIC1 is a new candidate tumor suppressor gene [23], but the relevance of its methylation in bladder cancer prognosis is still unknown. Although GSTP1 methylation is a well known event in the carcinogenesis of prostate cancer, its role in bladder carcinoma has yet to be defined. A recent study by Pljesa-Ercegovac and coworkers [24] revealed that high GSTP1 expression is associated with an altered apoptotic pathway and bladder cancer progression. As methylation reduces gene expression, our data are in agreement with those of Pljesa-Ercegovac, the absence of GSTP1 methylation observed in our study supporting the hypothesis of more aggressive behavior of bladder tumors and consequently of a higher relapse rate.

Although the role of RASSF1 in bladder cancer development is still unclear, Ha and coworkers reported that its methylation would seem to play a part in predicting recurrence in low grade and stage bladder tumors [25]. Surprisingly, we observed lower methylation levels of RASSF1 in recurrent tumors than in non recurrent ones, the discordance possibly due to different techniques used.

The MS-MLPA approach only permitted us to analyze one CpG site per probe, whereas several CpG sites may have been evaluated by Ha using the MS PCR technique [25]. For these reasons, we believe that further evaluation is needed to clarify the role of RASSF1 in bladder cancer, especially with regard to the correlation between its methylation status and protein expression.We also observed fairly low methylation frequencies for all the loci analyzed compared to those reported in other papers [26]. Such disagreement could, again, be due to the different analytical techniques adopted and/or to the different case series analyzed. Methylation cannot be the only mechanism of recurrence of NMIBC because the behavior of bladder tumors is fairly heterogeneous, as shown by Serizawa and coworkers [27] who observed an inverse correlation between FGFR mutations and hypermethylation events. In their study of the mechanisms of NMIBC recurrence, Bryan and coworkers [28], identified four reasons for relapse: incomplete resection, tumor cell re-implantation, growth of microscopic tumors and new tumor formation. These mechanisms differ greatly from each other and the identification of a single marker that is common to all four mechanisms appears improbable. It is more likely that a molecular marker characterizes tumor recurrence as a result of the third or fourth mechanisms, which may involve molecular alterations. This might explain why accuracy in our study only reached 72%.

## Conclusions

Our preliminary findings pave the way for in depth evaluation of the methylation levels of HIC1, GSTP1, and RASSF1 genes in larger case series to improve the clinical surveillance of patients with superficial bladder cancer.

## Consent

Written informed consent was obtained from the patient for the publication of this report and any accompanying images.

## Competing interests

The authors declare that they have no competing interests.

## Authors’ contributions

VC carried out the molecular genetic studies and drafted the manuscript; CM, DC, MT carried out the molecular genetic studies; RG, LS, FF participated in recruitment of patients and collection and assembly of data; CZ performed statistical analysis; RS helped to draft the manuscript and participated in the design of the study; DA and WZ participated in the design of the study and coordination. All authors read and approved the final manuscript.
